# Editorial: Insights in antigen presenting cell biology: 2021

**DOI:** 10.3389/fimmu.2022.1079913

**Published:** 2022-11-17

**Authors:** Peter van Endert

**Affiliations:** ^1^ Université Paris Cité, INSERM, CNRS, Institut Necker Enfants Malades, Paris, France; ^2^ Service Immunologie Biologique, AP-HP, Hôpital Universitaire Necker-Enfants Malades, Paris, France

**Keywords:** ERAP, immunopeptidome, antigen presentation, dendritic cell, macrophage, MHC class II, autophagy

The field of antigen presenting cell (APC) biology is commonly considered mature by immunologists, with most key mechanisms being explored and established in detail. Thorough understanding of APC biology has helped providing the fundaments, to name recent examples, for deciphering antiviral immune responses in the SARS-CoV2 pandemic, or to develop innovative tumor therapies targeting neoantigens. This notwithstanding, important gaps in our knowledge persist, perhaps foremost related to the biology of cells capable of cross-presenting exogenous antigens. For example, the important role of Wdfy4 in cross-presentation lacks explanation (1), as does the relative role of cross-dressing in priming antitumor responses (2). This collection of reviews and original papers highlights an array of issues in antigen presentation, ranging from broad discussions of the role of autophagy and of MHC-II processing in antigen presentation, over reviews of myeloid cell populations in lung and intestinal immunity and homeostasis, to more focused critical examination of enzymatic production of MHC-I ligands, discussing the controversial issue of splicing-derived neo-epitopes and the role of the ERAP2 aminopeptidase.

It is a coincidence that the collection comprises two articles dealing with ERAP2, an enzyme suddenly gaining a surprising prominence and public attention as this editorial is written. According to a very recent publication, expression of full length ERAP2, an aminopeptidase in the endoplasmic reticulum, may have provided significant protection against death from the bubonic plague in London and Denmark (3). This observation clearly comes as a major surprise since ERAP2, although in principle able to trim peptides for productive MIC-I presentation (4), has so far been considered of minor importance and certainly not key in the regulation of immune responses, as proposed somewhat enthusiastically in the recent paper. In fact, the literature concerning ERAP2 provides more open questions than answers concerning its function, as developed by Kusnierczyk. Why do 25% of the population lack proteolytically active ERAP2 protein? What is the role of the truncated isoform 3 produced upon viral infections? Does ERAP2 trimming of angiotensins regulate blood pressure in people? What underlies ERAP2 association with certain autoimmune diseases? And what is the role of the 30% proportion of ERAP2 forming heterodimers with ERAP1? Concerning the latter question, Papakyriakou et al. use molecular dynamics simulations to propose the energetically most likely structure of such dimers, opening an avenue for experimental testing through mutagenesis approaches. Given its recent link with protection against the black death, ERAP2 can be expected to be subject of vigorous research in the future.

In contrast to the presumably marginal role of ERAP2 in MHC-I ligand production, the key place of the proteasome in this context cannot be doubted. However, as discussed by Kloetzel, significant controversy surrounds a specific catalytic function of the proteasome, the generation of spliced peptides through ligation of two distant cleavage products. Following initial excitement upon the discovery that some tumor epitopes recognized by CD8+ T cells in patients were produced by splicing, a fairly controversial debate about the likelihood of such reactions and about the frequency of its products ensued. Contrasting with an early estimate of 25-30% of MHC-I ligands, more recent estimates assume much lower numbers, probably in the low to very low single figures. Whatever the percentage, focusing on the idea that production of neo-epitopes by splicing could produce promising targets of tumor therapy by adoptive T cell transfer, Kloetzel reports a sobering experience. After a massive experimental investment, two promising epitopes predicted by an algorithm turned out not to be generated and presented by tumor cells (5). Thus, low efficiency of splicing, as well as misleading conclusions from *in vitro* digestions with high substrate densities advise great caution with respect to spliced neo-epitopes and dampen hopes of exploitation for tumor therapy.

Non-controversial overviews of antigen processing pathways are given by Santambrogio and Münz. Santambrogio discusses the multiple factors determining the ligandome presented by MHC-II molecules, highlighting its plasticity. Although peptide ligands are foremost selected by highly polymorphic MHC-II molecules, factors such as metabolic or inflammatory stress can change the balance between cell-endogenous and exogenous ligands and their nature. The precise nature of the endocytic compartment where peptides are produced also affects the ligandome, as does the interplay between the peptide “editor” DM and its inhibitor DO that tilts towards stronger DM editing and a resulting focus on high-affinity epitopes as dendritic cells mature. Macro-autophagy is a well-established source of endogenous peptides presented by MHC-II molecules, however, as summarized by Münz, it also impacts MHC-I presentation through an enhancement of MHC-I internalization. Other non-canonical or less well-known impacts of autophagy proteins in antigen presentation include LC3-associated phagocytosis which enhances antigen uptake and degradation, degradation of intracellular compartments storing internalized antigens in dendritic cells, and extracellular release of vesicles containing autophagy receptors upon inhibition of autophagosome fusion with lysosomes.

Two reviews and one research paper discuss the antigen presentation role and identification of myeloid cells in three different tissue environments: intestine, lung and skin. Sasaki et al. provide an overview of models for deletion and tracking of type 1 conventional dendritic cells (cDC1) and discuss their role in intestinal immune homeostasis. Ablation of cDC1 specifically reduces T cells in the lamina propria and results in enhanced pathology in the model of DSS colitis, though not at steady state. The authors highlight the role of the cDC1-specific XCR1 receptor and its ligand XCL1 in the crosstalk between cDC1 and CD8+ T cells. Kawasaki et al. provide a broad review of the numerous myeloid but also epithelial cell types able to stimulate T cells in the lung. Type 1 and type 2 cDCs, monocytes, pDCs and type II alveolar cells all can present antigen to CD8 and/or CD4 T cells, but alveolar macrophages, the most numerous myeloid cell type in the lung, seem unable to do so. However, the macrophages are critical for lung homeostasis as they engulf not only cellular debris and material arriving through the airways but also surfactant which, in their absence, accumulates to cause lung proteinosis. Finally, Bourdely et al. demonstrate that autofluorescence can be used to identify highly phagocytic skin macrophages that accumulate around lesions and in squamous cell carcinoma. Autofluorescence, which can be measured best using a spectral flow cytometer, turns out to be a better marker of phagocytic activity than CD206 or Tim4, receptors for mannose and phopshatidyl serine, respectively.

## Author contributions

The author confirms being the sole contributor of this work and has approved it for publication.

## Conflict of interest

The author declares that the research was conducted in the absence of any commercial or financial relationships that could be construed as a potential conflict of interest.

## Publisher’s note

All claims expressed in this article are solely those of the authors and do not necessarily represent those of their affiliated organizations, or those of the publisher, the editors and the reviewers. Any product that may be evaluated in this article, or claim that may be made by its manufacturer, is not guaranteed or endorsed by the publisher.
